# Transcriptomic Profiling of Egg Quality in Sea Bass (*Dicentrarchus labrax*) Sheds Light on Genes Involved in Ubiquitination and Translation

**DOI:** 10.1007/s10126-017-9732-1

**Published:** 2017-02-08

**Authors:** Daniel Żarski, Thaovi Nguyen, Aurélie Le Cam, Jérôme Montfort, Gilbert Dutto, Marie Odile Vidal, Christian Fauvel, Julien Bobe

**Affiliations:** 1 Department of Lake and River Fisheries, University of Warmia and Mazury, ul. Oczapowskiego 2, 10-719 Olsztyn, Poland; 2 Department of Aquaculture, Szent István University, Páter Károly 1, Gödöllo, 2100 Hungary; 3 LPGP, INRA, 35000 Rennes, France; 4 IFREMER, UMR MARBEC (IRD, IFREMER, CNRS, Université de Montpellier), Station Expérimentale d’Aquaculture, Chemin de Maguelone, 34250 Palavas, France

**Keywords:** Microarray, Genomics, Transcriptomics, Aquaculture, Controlled reproduction

## Abstract

Variable and low egg quality is a major limiting factor for the development of efficient aquaculture production. This stems from limited knowledge on the mechanisms underlying egg quality in cultured fish. Molecular analyses, such as transcriptomic studies, are valuable tools to identify the most important processes modulating egg quality. However, very few studies have been devoted to this aspect so far. Within this study, the microarray-based transcriptomic analysis of eggs (of different quality) of sea bass (*Dicentrarchus labrax*) was performed. An Agilent oligo microarray experiment was performed on labelled mRNA extracted from 16 batches of eggs (each batch obtained from a different female) of sea bass, in which over 24,000 published probe arrays were used. We identified 39 differentially expressed genes exhibiting a differential expression between the groups of low (fertilization rate < 60 %) and high (fertilization rate > 60 %) quality. The mRNA levels of eight genes were further analyzed by quantitative PCR. Seven genes were confirmed by qPCR to be differentially expressed in eggs of low and high quality. This study confirmed the importance of some of the genes already reported to be potential molecular quality indicators (mainly *rnf213* and *irf7*), but we also found new genes (mainly *usp5*, *mem-prot*, *plec*, *cenpf*), which had not yet been reported to be quality-dependent in fish. These results suggest the importance of genes involved in several important processes, such as protein ubiquitination, translation, DNA repair, and cell structure and architecture; these probably being the mechanisms that contribute to egg developmental competence in sea bass.

## Introduction

One of the biggest obstacles in intensive aquaculture practice is highly variable and unpredictable gamete quality, with eggs being the highest concern (Bobe and Labbé 2010; Żarski et al. 2011). Up to now, there has been no clear husbandry protocol that could lead to the selection of spawners yielding high quality eggs. This is due to the great difficulty in identifying factors affecting egg quality in finfishes. This, in turn, is caused by the lack of clear and reliable egg quality indicators which, when available, could help in understanding the mechanisms determining eggs of high or low quality and identifying the traits which characterize “good” or “bad” spawners (Bobe and Labbé 2010; Żarski et al. 2012; Migaud et al. 2013).

By definition, egg quality is the ability of the egg to be fertilized and subsequently developed into a viable “normal embryo” (Bobe and Labbé 2010). The objective and precise evaluation of egg quality is one of the most important steps of the culture process, allowing the allocation/discarding of particular batches of eggs for/from further culture procedures (Migaud et al. 2013; Schaerlinger and Żarski 2015). Additionally, it enables the investigation of the effects of specific farming practices (e.g., the feeding regime of the broodstock, photothermal regimes applied, or the procedure of the induction of ovulation) on egg quality (Bromage et al. 1992; Brooks et al. 1997; Bobe and Labbé 2010). This has caused the development of several biological and biochemical indicators of egg quality, allowing the general discrimination of eggs of high or low quality. These include, among others, blastomere morphology (Bobe and Labbé 2010), oil droplet distribution (Mansour et al. 2007), oil droplet fragmentation (Żarski et al. 2011), cortical reaction intensity (Żarski et al. 2012), egg swelling intensity (Lahnsteiner et al. 2001), pH and electrical conductivity of the ovarian fluid (Lahnsteiner et al. 1999, 2001; Skaalsvik et al. 2015), causing the turbidity of water by the batch of eggs (Mansour et al. 2007), the floating ability of pelagic eggs (Carrillo et al. 1989), as well as egg nutritional constituents, including fatty acid profiles (Lahnsteiner et al. 1999; Henrotte et al. 2010). Most of these indicators are, however, valid only in particular species or under particular farming conditions (Ciereszko et al. 2009) or both. Therefore, the only generally reliable true assessment of egg quality may still be performed at different steps of embryonic development to evaluate developmental success and conformity.

The transcriptomic profile of eggs is one of the emerging tools allowing the improvement of husbandry practices in aquaculture (Bonnet et al. 2007; Bobe and Labbé 2010; Mommens et al. 2014; Chapman et al. 2014). Considering that the entire oogenesis in finfishes is a long-lasting process during which the oocytes are subjected to a number of morphological, biochemical, and molecular changes, the final characteristics of an ovulated egg are a specific “summary” of the oogenesis course (Lubzens et al. 2010; Chapman et al. 2014). This also applies to transcriptomic profiles, which were found to significantly differ between particular phases of oogenesis (Lanes et al. 2013) and in response to different life history traits or different husbandry practices (Bonnet et al. 2007). Therefore, the structure of maternally inherited messenger RNAs (mRNAs) contained in the egg upon ovulation can be considered as a result of previous events (Gohin et al. 2010). Until now, only a few studies have focused on the transcriptomic profile of ovulated eggs and its relation to the developmental competence of the eggs. This research was performed in rainbow trout, *Oncorhynchus mykiss* (Walbaum) (Aegerter et al. 2005; Bonnet et al. 2007), Atlantic halibut, *Hippoglossus hippoglossus* (L.) (Mommens et al. 2010, 2014), Atlantic cod, *Gadus morhua* L. (Lanes et al. 2012; Rise et al. 2014), and recently in striped bass, *Morone saxatilis* (Walbaum) (Chapman et al. 2014).

To investigate the molecular determinants of egg quality in finfishes, highly expressed genes with the highest differences in expression value (fold change) between eggs of high and low quality were searched for. It is generally agreed that the expression of a single gene is unlikely to be a reliable egg quality indicator valid in a wide range of situations. Some authors have already indicated that at the molecular level egg quality is probably characterized by a suite of genes for which variation and/or a particular pattern expression would reveal the real transcriptomic profile of a “good” and/or “bad” egg (Chapman et al. 2014; Rise et al. 2014; Sullivan et al. 2015). To this end, further large scale transcriptomic studies are required to identify the best candidate genes, presumably contributing to our better understanding of the molecular mechanisms standing behind the developmental competence of eggs.

Sea bass, *Dicentrarchus labrax*, is one of the most important cultured species in the Mediterranean Sea basin (Shields 2001). Its aquaculture has been constantly developing for the last 30 years, and a major production increment was observed within the last two decades. From the very beginning, controlled reproduction was one of the most important constraints and was therefore intensively studied in this species (e.g., Mylonas et al. 2003), while egg quality was specifically investigated in several studies (Devauchelle and Coves 1988; Carrillo et al. 1989; Cerdá et al. 1994, 1995). The protein composition (Carnevali et al. 1998) of eggs of varying quality, as well as comparable proteomic analysis of eggs obtained from domesticated and wild females (Crespel et al. 2008), was also studied in sea bass. To date, there has been no large scale (i.e., genome wide) transcriptomic analysis of the eggs of this species characterized by different quality.

In the present study, the comparative analysis of the transcriptomic profile (microarray analysis of total mRNA obtained from 16 egg batches) of sea bass eggs, obtained after the application of standardized reproductive protocol, and biological egg quality was performed. Additionally, the relative abundance of the mRNAs of eight genes was verified by quantitative PCR analysis. Gene ontology analysis of differentially expressed genes was performed to identify the main biological processes regulated at the transcriptomic level, as being possibly related to egg quality.

## Material and Methods

### Artificial Spawning and Fertilization

For the experiment, 48 females of cultured European sea bass females (with average weight of 2.90 kg ± 1.1 SD) were reproduced at IFREMER experimental station of Palavas, France (experimentation agreement C34-19266), and resulting from a standard protocol including female stimulation, artificial spawning, artificial fertilization, and incubation of the embryos as follows. During the spawning season, female genital state was regularly controlled through the observation of an ovarian biopsy. The maturation stage of each female was determined according to Fauvel and Suquet (1998). Only females at postvitellogenic stage were chosen for further procedures and subject to intramuscular (at the dorsal musculature, behind the dorsal fin) injection of LHRHa (D-Trp^6^, SIGMA) solution at a dose of 10 μg kg^−1^.

After induction, females were individually kept indoor in 1-m^3^ tanks at a temperature of 12.9–13.5 °C, salinity of 34.8–36.2 ppt, pH of 7.9–8.1, and photoperiod of 8/16 (light/dark). After ovulation (occurring between 68 and 75 h following hormonal injection), the eggs from each female separately were stripped exhaustively into dry containers the time after stimulation and the total volume of the collected egg batch was recorded. Then, eggs were dispatched into aliquots for different treatments. One portion of 500 μl (approx. 500 eggs) was deep frozen in duplicate in liquid nitrogen and stored at −80 °C until molecular analysis. Batches of 5 ml of eggs were fertilized in triplicates with an aliquot of cryopreserved sperm. For that purpose, a mixture of sperm obtained from 20 males was dispatched into straws and cryopreserved according to the method of Fauvel et al. (1998) what allowed to cover the needs for all the crosses in order to exclude possible paternal effect in the analysis of embryonic development course (Saillant et al. 2001). During fertilization, the amount of sperm was always adjusted to 2 · 10^5^ of spermatozoa per egg, as recommended by Fauvel et al. (1999). Fertilization was performed with seawater as an activating solution.

### Investigation of the Early Development of Embryos and Larvae and Determination of Egg Biological Quality

Just after the fertilization (approx. 2 min), aliquots of 2.5 ml of eggs from each female were transferred into beakers and incubated in seawater at 14 °C for 3 h. After that period, the second cleavage was already completed and all the developing embryos had reached the four-cell stage. At this stage (3 h post-fertilization, HPF), developing and non-developing eggs were counted allowing first estimation of fertilization rate. From each batch, 72 developing eggs were separated and dispatched into microwell plates as described by Panini et al. (2001) in order to determine the developmental success of each egg separately after 3 and 4 days of incubation (3 and 4 days post-fertilization, DPF) as well as hatching rate (5 DPF). The incubation was conducted in seawater at a temperature ranging between 13.3 and 14.4 °C and with salinity of 35.7–36.1 ppt.

For the transcriptomic analysis, 16 egg batches were chosen, among which 8 batches represented high and 8 batches low egg quality groups. Each group was chosen taking into account two evaluation steps. The first step involved morphological evaluation of the external appearance of the eggs aimed at avoiding taking for further analysis the ones characterized by extreme abnormalities such as overripening signs. This allowed to consider for further analysis egg batches having “normal” morphology (as it is typically performed in aquaculture practice). Next, the choice of the egg samples for further analysis was based on evaluation of developmental competence of the eggs, where finally fertilization rate at 3 HPF, with 60 % of fertilization rate considered as the threshold value between the groups, was used. This threshold was chosen, however, on the basis of the developmental competence observed throughout embryonic development and hatching. For example, the neighboring samples (L7 and H1) were characterized by ∼10 % differences in fertilization rate but also over 25 % in hatching rate. The differences between the groups were checked with Mann-Whitney *U* test (Statistica, v.12, StatSoft, USA) at a significance level of 5 % (*p* < 0.05).

### RNA Extraction

RNA extraction was performed with the use of TRI Reagent as previously described for rainbow trout (Bonnet et al., 2007). Briefly, the RNA from 16 batches of eggs was extracted each time from about 0.2 ml of eggs by using 3 ml of TRI Reagent. A NanoDrop® NP-1000 spectrophotometer (NanoDrop Technologies, Wilmington, DE, USA) was used to measure the quantity of the RNA samples. The quality of the RNA samples was checked using a 2100 Bioanalyzer (Agilent Technologies Inc., Santa Clara, USA), and samples with RIN value higher than 8.5 were used for further analysis.

### Microarray Analysis

Sea bass gene expression profiling was conducted using an Agilent 8x60K high-density oligonucleotide microarray (GEO platform no. GPL22152). Labeling and hybridization steps were performed following the “One-Color Microarray-Based Gene Expression Analysis (Low Input Quick Amp labeling)” Agilent protocol. Briefly, for each sample, 150 ng of total RNA was amplified and labeled using Cy3-CTP. Yield (>1.65 μg cRNA) and specific activity (>9 pmol of Cy3 per μg of cRNA) of Cy3-cRNA produced were checked with the Nanodrop. Of Cy3-cRNA, 1.65 μg was fragmented and hybridized on a sub-array. Hybridization was carried out for 17 h at 65 °C in a rotating hybridization oven prior to washing and scanning with an Agilent Scanner (Agilent DNA Microarray Scanner, Agilent Technologies, Massy, France) using the standard parameters for a gene expression 8x60K oligoarray (3 μm and 20 bits). Data were then obtained with the Agilent Feature Extraction software (10.7.1.1) according to the appropriate GE protocol (GE1_107_Sep09) and imported into GeneSpring GX software (Agilent Technologies, Santa Clara, CA, USA) for analysis. Data were published at the NCBI’s Gene Expression Omnibus (Edgar et al. 2002) and are accessible through GEO series accession number GSE84559. Samples were randomly distributed on the microarray for hybridization. The gene expression data was scale normalized and log(2) transformed before the statistical analysis.

Differentially expressed genes between the groups representing high (samples H1–H8) and low (samples L1–L8) egg quality were identified with the use of GeneSpring GX software. Due to the hybridization failure of two egg samples (one sample representing low and one sample representing high egg quality) for further analysis of the microarray data, only 14 samples were taken into account. The differences between the groups were analyzed with unpaired *t* test after application of minimum twofold change filter with the significance level of 5 % (*p* < 0.05). Next, normalized log(2)-transformed expression values as well as raw signal data were exported to MS Excel software where they were filtered according to the raw signal, i.e., only the genes for which raw signal of more than 50 % samples was above 50 was taken for further analytical steps. Average linkage clustering analysis (Gene Cluster 3.0) was performed for the differentially abundant genes (unsupervised linkage) and in relation to egg quality (supervised linkage).

### Reverse Transcription and Real-Time qPCR

Reverse transcription PCR was performed with the Maxima® First Strand cDNA Synthesis Kit (Thermo Scientific) according to the manufacturer’s protocol. Briefly, 2 μg of total RNA was mixed with 4 μl of 5X Reaction Mix and 2 μl Maxima Enzyme Mix. Next, the content of each tube was filled up to 20 μl with nuclease-free water. The samples were then gently mixed, centrifuged, and incubated for 10 min at 25 °C followed by 30 min at 50 °C. The reaction was terminated at 85 °C for 5 min.

Real-time qPCR was performed using the Step One Plus system (Applied Biosystems, Foster City, USA). Reverse transcription products were diluted to 1:20 and 4 μl was used for real-time PCR, using GoTag® qPCR Master Mix (Promega, Madison, WI, USA) and 1500 pmol of each primer (Table 1). The enzyme was activated 2 min at 95 °C, followed by 40 cycles of denaturation at 95 °C for 15 s, and annealing and elongation at 60 °C for 1 min. After amplification, a melting curve was performed according to the manufacturer’s recommendations to check the amplification specificity. Relative expression was normalized using the geometric mean of expression values recorded by qPCR of the six genes exhibiting the most stable expression level. For each sample, the expression level of each gene was analyzed in triplicates. Finally, the data between the groups (representing low and high egg quality) were analyzed with the Mann-Whitney *U* test (Statistica, v.12, StatSoft, USA). Differences between groups were considered significant when *p* < 0.05. With real-time qPCR, 8 genes for all the 16 samples were analyzed.

**Table 1:** The primer sequences used for real-time qPCR of RNA obtained from the eggs of European sea bass characterized by different quality

Symbol	Forward sequence	Reverse sequence
Differentially expressed genes		
*polk*	GCAAAGAAACTCTGCCCCAA	CCGGATCTCATTGCACACAG
*rab2a*	GGCTTTCATCAACACAGCCA	GGAGTTGGTGGTAGGATGCT
*irf7*	CTAACCGTCTCCAGCTCCAT	CAGTGGATGGGAAGCAGAGA
*usp5*	AGAAGGATCAGCAGTGGGTC	GCTCCCTCCCTTGTCTCATT
*kctd12*	GGAGTGTGCTTGCATTGTCA	CTTATCCACCACACCCCTCA
*plec*	TCCTCTCAGTCCAGCAAAGG	ATCCTGTCCTTGAGCCAGTC
*hy-prot(1)*	GGTAGCCCTGCCCTTCTTAA	TTAGTGGGCTGCATCCTCAA
*mem-prot*	ACGTTCTGTCGTGCCTCTCT	TGCAGAGGGCTTTTGCTATT
Most stable genes		
*hspa14*	GTGCCTGAGGAAGAGTCTGT	CCCGATGGAAGGAGAACAGT
*cops3*	GCCTTGGAGCAGTTTGTGAA	TTGATCAACTCGCACAGCTG
*mtif3*	CACCATGCACCGTCTAGTTG	TTTGTGTTCACTGACCAGCG
*eif3ea*	CTCACCACCAAAATTGCCCA	GCGAAGTCCACCATGTTTGT
*hectd3*	AGCCGCACTCAAAGGAAAAG	CAGAGTCTACAGCGGGGAAA
*eif3e*	AGAGCACCAAGAACGAGACA	TCCATCTTGCTTGAGTCGCT

Differentially expressed genes refer to the ones exhibiting different mRNA abundance between the groups representing “low” and “high” egg quality. Most homogenized genes are the ones used for the data normalization. For each gene symbol, the protein is provided according to the iHOP database or specific abbreviation was introduced (explained in the footnote of the table). Numbers in parenthesis represent number of subsequent gene, when more than one gene with the same name was identified

*hy-prot* hypothetical protein, *un-prot* uncharacterized protein, *n-f* protein was not found, *mem-prot * membrane protein

### Data Mining

All the differentially expressed genes as well as genes which expression level highly correlated with the fertilization rate were identified using Basic Local Alignment Search Tool (BLAST). For this purpose, expressed sequence tag (taken for the microarray design) search was performed with standard nucleotide BLAST tool (BLASTN) followed by translated BLAST tool (BLASTX). The official gene symbol ontology of the identified genes was obtained using the UniProt Knowledgebase (UniProtKB).

## Results

### Biological Quality of Eggs

**Fig. 1:**
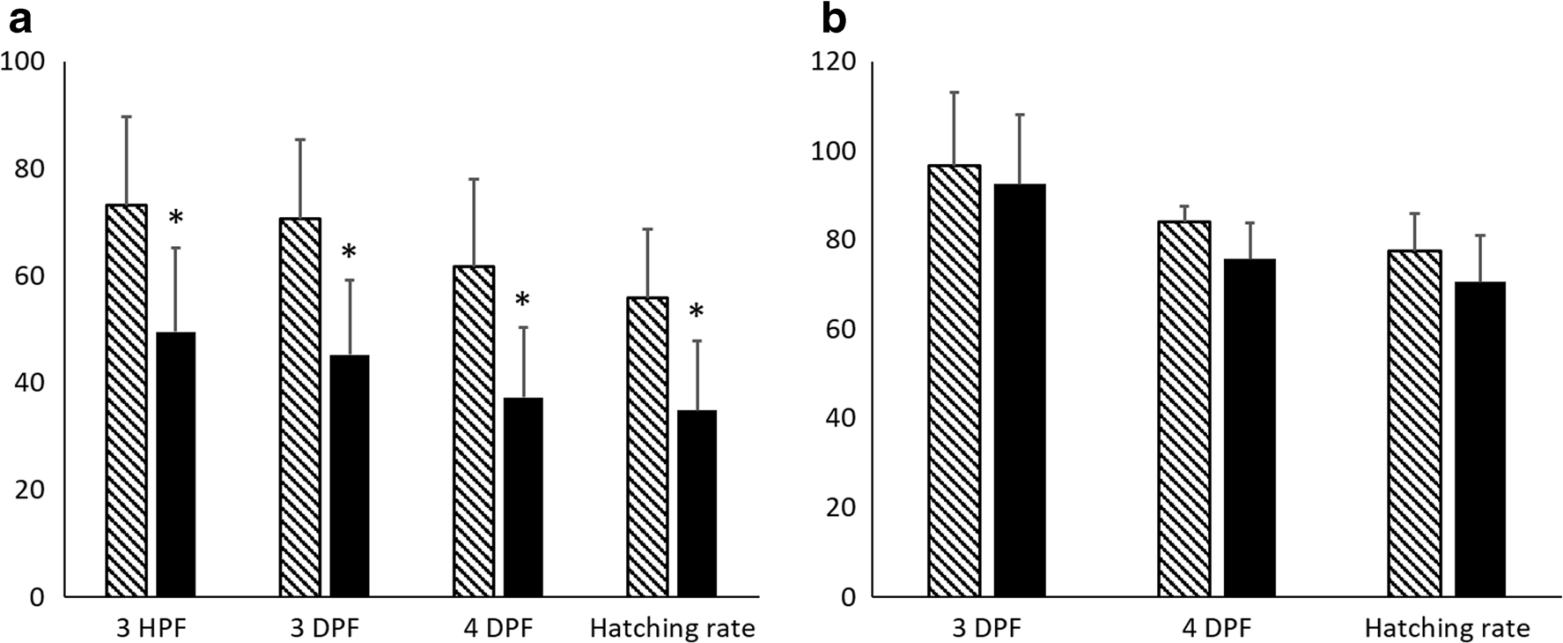
Fertilization (*HPF* hours post-fertilization, *DPF* days post-fertilization) and hatching rates of embryos of European sea bass representing high (*gray bars*) and low (*black bars*) egg quality. **a** Fertilization and hatching rate was calculated taking into account all the eggs used for fertilization from each batch. **b** Fertilization and hatching rate was calculated only for the eggs which started to develop at third HPF. Data marked with *asterisk* were statistically different (*p* < 0.05)

The mean fertilization rate observed at 3 HPF in the group characterized by low egg quality was significantly lower (*p* < 0.05) than in the group representing high egg quality. This was observed throughout development until hatching, where significant differences between the groups were also observed (Fig. 1a). The fertilization and hatching rates, when only fertilized eggs (the first cell cleavages were noticed at 3 HPF) were taken for further incubation, did not reveal any differences in terms of the developmental competence of the embryos up to the larval stage (Fig. 1b). The results of the biological evaluation of egg quality confirmed the relevance of the estimation of the fertilization rate at 3 HPF, which was then considered as the quality indicator used for the identification of groups of high and low egg quality.

### Microarray Analysis

**Fig. 2:**
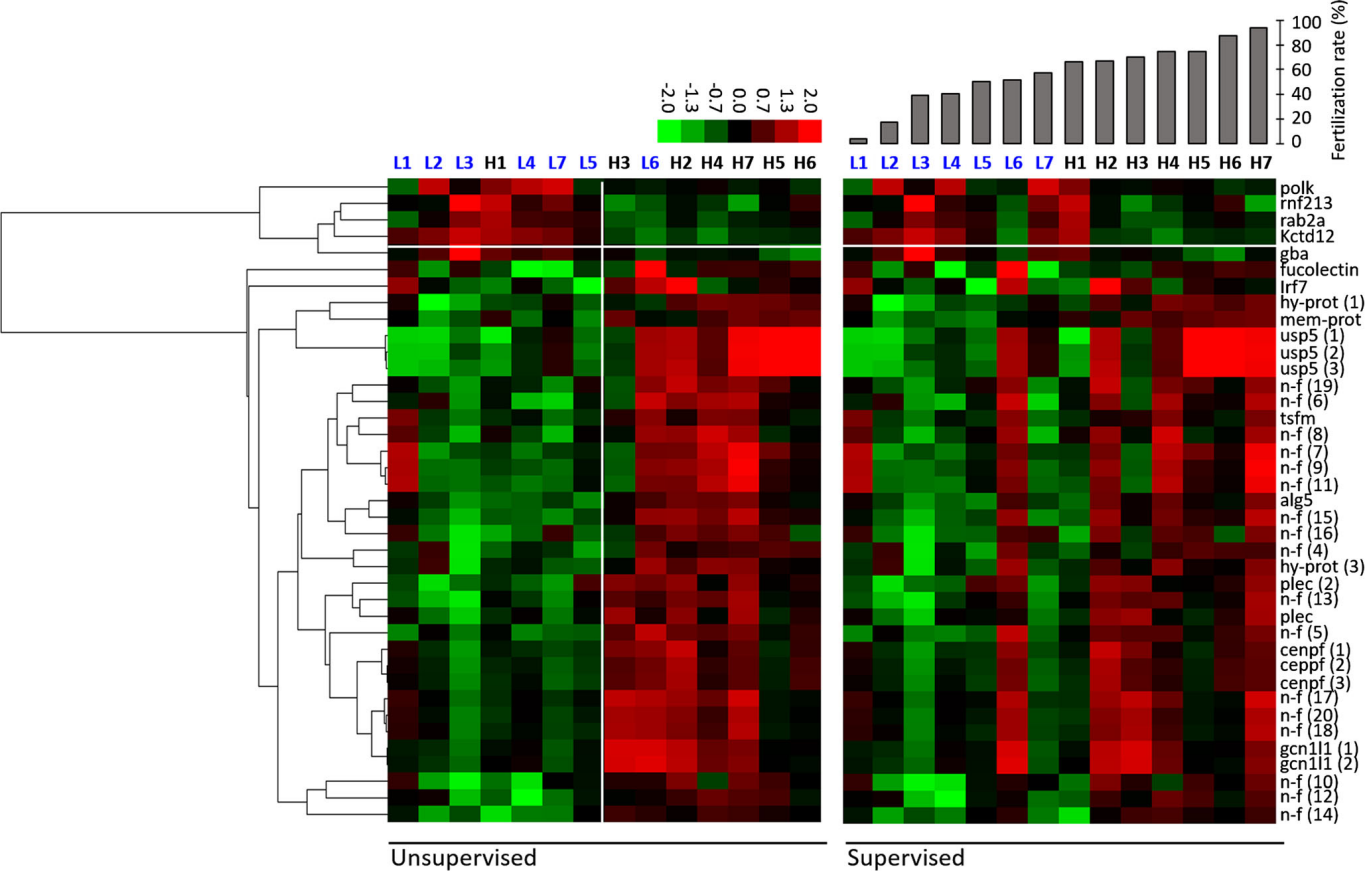
Unsupervised (*on the left*) and supervised (*on the right*) average linkage clustering of 39 differentially expressed genes. Supervised clustering was performed according to the fertilization rate (diagram above the respective samples) recorded 3 HPF. Each *row* represents the same gene with symbol given on the right-hand side. Each *column* represents a RNA sample with its symbol provided above the respective column. Samples annotated with *black font* (H1–H7) represented high quality group, whereas samples annotated with *blue font* (L1–L7) represented low egg quality group. Expression level for each gene is presented using color intensity scale, where *red* and *green* represents over- and under-expression levels, respectively. *Black color* represents median abundance of the gene

The microarray analysis resulted in the identification of 461 differentially expressed genes between the groups representing low and high egg quality. However, in only 39 genes more than 50 % of the samples exhibited raw signal above 50. Therefore, only those 39 genes were considered in further analysis. Unsupervised average linkage clustering revealed four clusters distinguishing groups of low and high egg quality, as well as under- and over-expression patterns of the analyzed genes. Two samples were clustered into not originally assigned groups, i.e., sample L6, initially assigned to the group of low egg quality, was clustered together with the high egg quality samples. Similarly, sample H1, which was originally assigned to the high egg quality group, was clustered together with low egg quality samples (Fig. 2). Supervised (according to the fertilization rate) average linkage clustering of the same genes clustered genes in the same order (Fig. 2). Three samples characterized by the lowest (L1–L3) and four by the highest quality (H4–H7) were grouped at the extreme edges of the cluster, being therefore in agreement with the unsupervised linkage. Among the samples characterized by moderate quality (between 50 and 75 % of FR), changes in positioning were observed, in comparison to unsupervised linkage, with only two samples (L6 and H1) changing their position for more than two (Fig. 2). Descriptions and ontology of all the differentially expressed genes are presented in Table 2.

**Table 2:** Gene symbol, description, GenBank number (or NCBI Reference Sequence number for the PREDICTED proteins), and Gene Ontologies (with UniProtKB accession no.) of all the genes found to be differentially expressed (on the base of microarray analysis) when groups of “high” and “low” egg quality were compared, genes which were highly correlated with the egg fertilization rate as well as the most homogenized genes (used for qPCR data normalization)

Symbol^a^	Description^b^	GenBank accession no.	GenBank/NCBI reference sequence	UniProtKB/Swiss-Prot Gene Ontology			UniProtKB accession no.
				function	Process	Component	
Differentially expressed genes							
*polk*	DNA polymerase kappa	FM009023	KKF10166	DNA binding, DNA-directed DNA polymerase activity, transferase activity	DNA repair, translesion synthesis, nucleotide-excision repair, DNA gap filling	Nucleus, nucleoplasm	Q9UBT6
*rnf213*	E3 ubiquitin-protein ligase RNF213	FM016314	XP_008298790	Ubiquitin-protein transferase activity, protein binding	Protein ubiquitination	Cytoplasm, nucleolus, membrane	Q63HN8
*rab2a*	Ras-related protein Rab-2A	FM000715	XP_008332898	GTPase activity, protein binding, GTP binding	Mitotic cell cycle, intracellular protein transport, metabolic process	Nucleus, Golgi membrane, endoplasmic reticulum, membrane	P61019
*kctd12*	BTB/POZ domain-containing protein KCTD12	FM024677	XP_008308481	Poly(A) RNA binding	Regulation of G protein-coupled receptor protein signaling pathway, protein homooligomerization	Plasma membrane, presynaptic membrane, synapse	Q96CX2
*gba*	Glucosylceramidase	FM021788	XP_010748685	Glucosylceramidase activity, receptor binding, hydrolase activity	Response to hormones (testosterone, estrogen, glucocorticoid) and pH, metabolic process (lipid, carbohydrate, sphingolipid, glycosphingolipid)	Lysosome membrane, lysosome lumen	P04062
*plec(2)*	Plectin-like isoform X5	FM008272	XP_011603659	Protein binding, poly(A) RNA binding	Cellular component disassembly involved in execution phase of apoptosis, programmed cell death, apoptotic process	Cytoplasm, plasma membrane	Q15149
*plec(1)*	Plectin-like isoform X3	FM004961	XP_010767028	Protein binding, poly(A) RNA binding	Cellular component disassembly involved in execution phase of apoptosis, programmed cell death, apoptotic process	Cytoplasm, plasma membrane	Q15149
*usp5(1)* *usp5(2)* *usp5(3)*	Ubiquitin carboxyl-terminal hydrolase 5	FM016194	XP_010733307	Ubiquitin-specific protease activity, protein binding, metal ion binding, hydrolase activity, cysteine-type peptidase activity	Ubiquitin-dependent protein catabolic process, positive regulation of proteasomal ubiquitin-dependent protein catabolic process, protein K48-linked deubiquitination	Lysosome	P45974
*cenpf(1)* *cenpf(2)* *cenpf(3)*	Centromere protein F (No. 1)	FM016762	XP_010730622	Protein binding, protein homodimerization activity, transcription factor binding	Regulation of G2/M transition of mitotic cell cycle, chromosome segregation, metaphase plate congression, kinetochore assembly, regulation of striated muscle tissue development	Chromosome centromeric region, kinetochore, nucleus, cytosol	P49454
*mem-prot*	–	FM024907	WP_038038408	n/a	n/a	n/a	n/a
*hy-prot(3)*	–	FM013574	WP_033116978	n/a	n/a	n/a	n/a
*hy-prot(1)*	–	FM005046	WP_030404274	n/a	n/a	n/a	n/a
*tsfm*	TSFM Ts translation elongation factor, mitochondrial	FM024343	KKF32528	Translation elongation factor activity	Translational elongation	Mitochondrion	B5X5B4
*irf7*	Interferon regulatory factor 7	FM005989	KKF30244	Protein binding, DNA binding, RNA polymerase II core promoter proximal region sequence-specific DNA binding transcription factor activity	Regulation of adaptive immune response, regulation of transcription, DNA-templated, regulation of innate immune response	Nucleus, cytoplasm	P70434
*gcn1l1(1)* *gcn1l1(2)*	Translational activator GCN1 (No. 1)	FM024347	KKF24933	Translation factor activity, RNA binding	Regulation of translation	Cytoplasm, ribosome, membrane	Q92616
*fucolectin*	Fucolectin-4	FM025664	KKF16053	Fucose binding, carbohydrate binding, calcium ion binding, metal ion binding	Regulation of complement activation, lectin pathway, regulation of cellular defense response, regulation of innate immune response	Extracellular region	Q9I928
*alg5*	Dolichyl-phosphate beta-glucosyltransferase	FM024334	KKF15163	Transferase activity, transferring glycosyl groups	Protein glycosylation, post-translational protein modification, transferase activity	Endoplasmic reticulum, endoplasmic reticulum membrane	Q9Y673
*n-f(4)*	–	FM024309	–	–	–	–	–
*n-f(5)*	–	FM027632	–	–	–	–	–
*n-f(6)*	–	FM028757	–	–	–	–	–
*n-f(7)*	–	FM019745	–	–	–	–	–
*n-f(8)*	–	FM015144	–	–	–	–	–
*n-f(9)*	–	FM021366	–	–	–	–	–
*n-f(10)*	–	AM984356	–	–	–	–	–
*n-f(11)*	–	FM021366	–	–	–	–	–
*n-f(12)*	–	FM007989	–	–	–	–	–
*n-f(13)*	–	FM000334	–	–	–	–	–
*n-f(14)*	–	FM002847	–	–	–	–	–
*n-f(15)*	–	FM010821	–	–	–	–	–
*n-f(16)*	–	FM001796	–	–	–	–	–
*n-f(17)*	–	FM014863	–	–	–	–	–
*n-f(18)*	–	FM014863	–	–	–	–	–
*n-f(19)*	–	FM020519	–	–	–	–	–
*n-f(20)*	–	FM014863	–	–	–	–	–
Genes exhibiting the most stable expression level							
*hspa14*	Heat shock 70 kDa protein 14	FM023807	KKF23225	Protein and ATP binding	“De novo” cotranslational protein folding	Cytoplasm, cytosol, membrane, ribosome	Q0VDF9
*cops3*	COP9 signalosome complex subunit 3	FM027858	XP_008300965	Protein binding	Ubiquitin-dependent protein catabolic process, in utero embryonic development, signal transduction, protein binding	Nucleus, cytoplasm	Q9UNS2
*mtif3*	Translation initiation factor IF-3, mitochondrial	FM022743	KKF15983	Translation initiation factor activity	Mitochondrial translational initiation	Mitochondrion	Q9H2K0
*eif3ea*	Eukaryotic translation initiation factor 3 subunit E-A	FM026913	KKF20815	Translation initiation factor activity	Regulation of translational initiation	Nucleus, cytoplasm	Q6DRI1
*hectd3*	E3 ubiquitin-protein ligase HECTD3	FM020929	KKF23624	Ubiquitin-protein transferase activity	Protein ubiquitination involved in ubiquitin-dependent protein catabolic process	Cytoplasm	Q3U487
*eif3e*	Eukaryotic translation initiation factor 3 subunit E	FP237239	XP_010738667	Translation initiation factor activity, protein binding	Regulation of translational initiation	Nucleus, cytoplasm, cytosol	P60228

Numbers in parenthesis represent number of subsequent gene, when more than one gene with the same name was identified

Specific, not described symbols: *n-f* protein was not found, *hy-prot* only sequence of hypothetical protein could be found, *mem-prot* refer to unspecified membrane protein, *un prot* only sequence for uncharacterized protein was found

^a^For all the genes, when possible, gene symbol identical to human one was given

^b^For all the genes, when possible, gene description identical to human one was given

### Real-Time qPCR Analysis

**Fig. 3:**
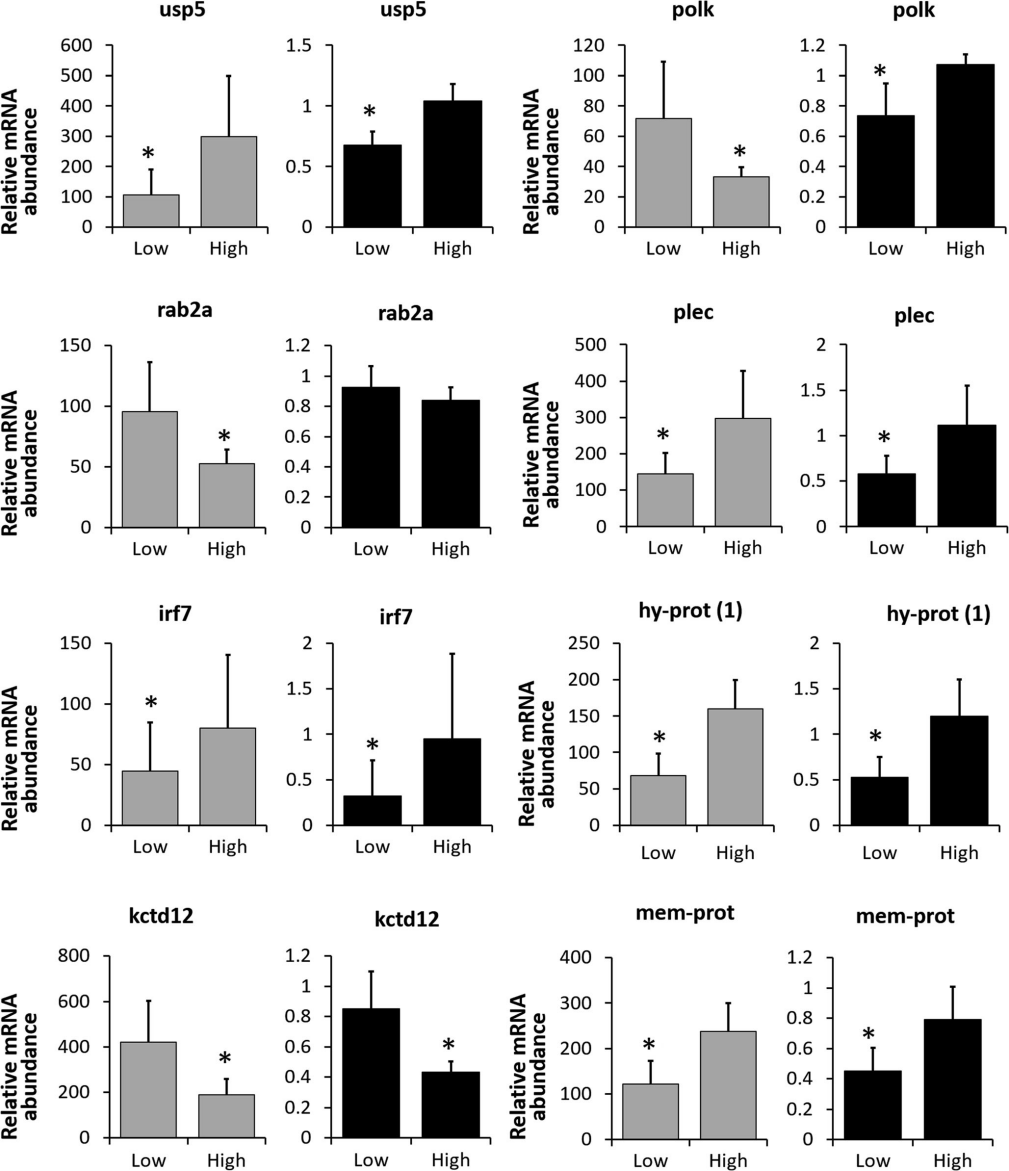
The relative gene expression level in “low” and “high” egg quality groups obtained after microarray (*gray bars*) and real-time qPCR (*black bars*) analysis. Data marked with an *asterisk* were statistically different (*p* < 0.05). Description and ontology of all the genes are presented in Table 2

Among the eight genes chosen for the real-time qPCR analysis, significant differences in relative expression between the low and high egg quality groups were recorded in seven of them. However, for one gene—DNA polymerase kappa (*polk*)—qPCR revealed the opposite expression pattern from the one found during the microarray analysis (Fig. 3). For ras-related protein Rab-2A (*rab2a*), qPCR did not confirm significant differences between the experimental groups. Descriptions and ontology of all the analyzed genes are presented in Table 2.

### Genes Exhibiting the Most Stable Expression Level

**Fig. 4:**
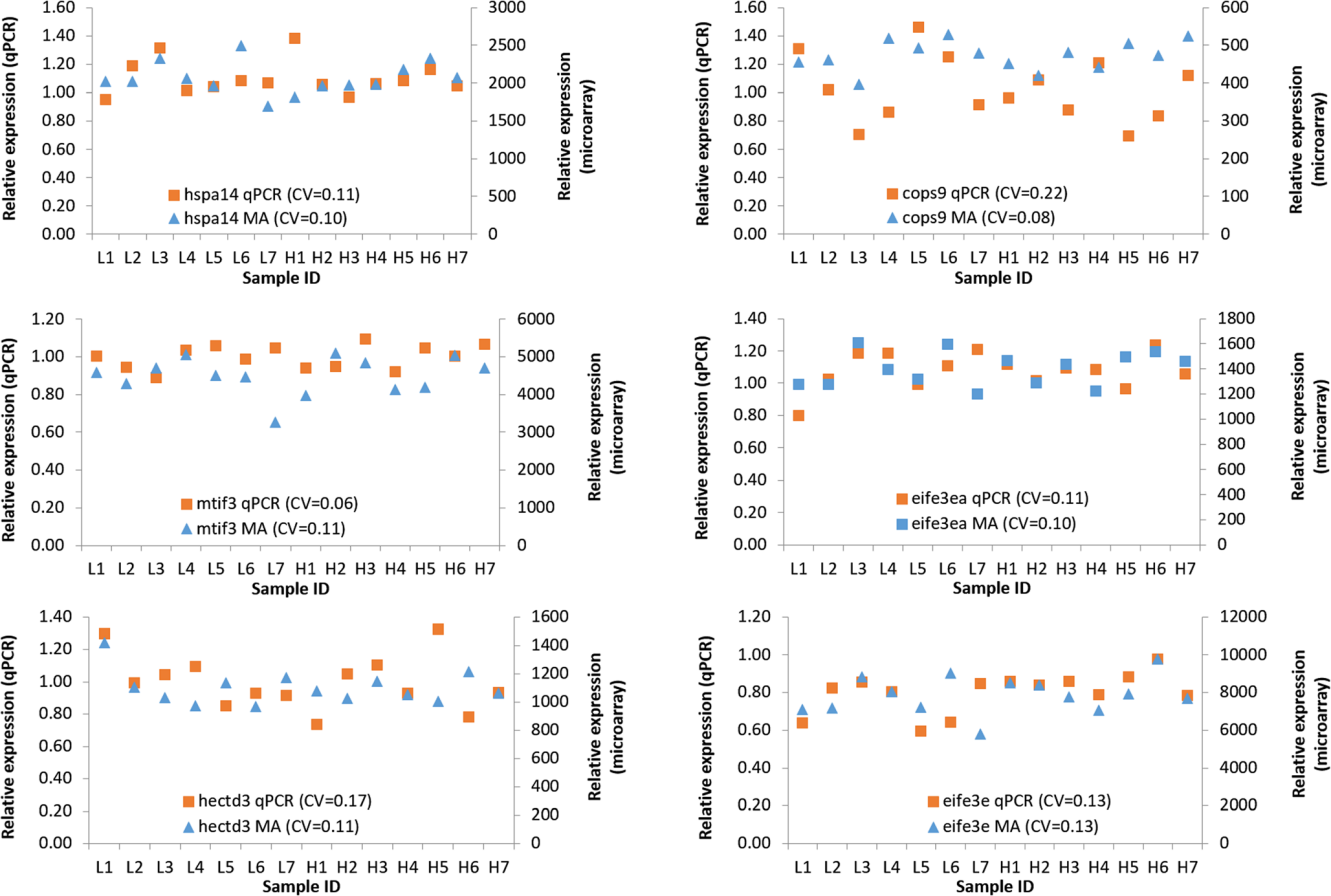
Relative expression level of the most stable genes (which were used for data normalization) recorded during the microarray (*MA*) analysis and qPCR analysis. For expression level of each gene, coefficient of variation (*CV*) was calculated and provided. Description and ontology of all the genes are given in Table 2. Sample ID represents egg samples assigned to different quality groups (L1–L7 for low egg quality group, H1–H7 for high egg quality group)

The real-time qPCR of genes exhibiting the most stable expression level during the microarray analysis revealed similar expression patterns and stability in all the six analyzed genes. Only in the case of COP9 signalosome complex subunit 3 (*cops9*) did the coefficient of variation after qPCR exhibits an over 10 % higher value as compared to the microarray data. On the other hand, the results of qPCR analysis of mitochondrial translation initiation factor IF-3 (*mtif3*) indicate that expression level was more stable than that recorded during the microarray analysis (Fig. 4). Nonetheless, the qPCR validation confirmed the stability of those genes, which were subsequently used for qPCR data normalization.

## Discussion

### Biological Egg Quality

Biological egg quality in finfishes is classically assessed through the monitoring of embryonic development at different stages and/or at hatching (Forniés et al. 2001; Żarski et al. 2011, 2012). For many fish species, the hatching rate has been considered more reliable than embryonic survival at earlier stages (Żarski et al. 2011; Schaerlinger and Żarski 2015). This is due to successive embryo mortality during incubation, which can be associated with impairment of important events occurring during embryogenesis, such as, for example, activation of the zygotic genome or the exhibition of serious developmental abnormalities (e.g., Schaerlinger and Żarski 2015). However, it should be highlighted that poor egg quality is very often manifested during early embryonic development (Chapman et al. 2014), which enables the use of early developmental stages (such as, e.g., the eight-cell stage) as a reliable quality indicator (Mommens et al. 2014). The data obtained in our study clearly indicate that embryonic survival at 3 HPF was reliable enough to estimate the overall quality of eggs in sea bass. This suggests that eggs without any developmental competence failed to develop at the earliest possible stage. A similar observation was also reported for striped bass, for which comparable survival of viable embryos was recorded up to 5 days post-hatch, even if originated from batches characterized by different quality (Chapman et al. 2014). In the case of sea bass, it was also previously reported that determination of embryonic survival was a reliable quality indicator (Saillant et al. 2001). It can thus be concluded that, in the case of sea bass, the embryos exhibiting high developmental competence at the earliest embryonic stages exhibited similar developmental competence later on.

### Differences Between the Groups of High and Low Quality

The results of our study clearly suggest the possible importance of genes responsible for intracellular protein degradation in the formation of egg developmental competence. One of the genes is ubiquitin carboxyl-terminal hydrolase 5 (*usp5*), whose expression was significantly higher in high quality eggs (confirmed also by qPCR). It encodes one of the deubiquitinating enzymes (DUB), which are enzymes involved in ubiquitin-mediated proteolysis of proteins (Nandi et al. 2006; Dayal et al. 2009). Through their important function, DUBs play a key regulatory role in many processes, from hereditary cancer to neurodegeneration (Nijman et al. 2005). Interestingly, another ubiquitin-related gene, E3 ubiquitin-protein ligase RNF213 (*rnf213*), encoding a probable ligase catalyzing the final phase of the formation of protein-ubiquitin complex (e.g., Liu et al. 2011), was upregulated in eggs of low quality, which is in accordance with the quality-related expression pattern of *rnf213* reported for Atlantic halibut (Mommens et al. 2014). The mRNA level of the other DUBs (e.g., ubiquitin-specific peptidase 11 and 14—*usp11* and *usp14*, respectively), as well as other genes related to the ubiquitin-related processes, was also reported to be positively correlated with egg quality in striped bass (Chapman et al. 2014). This suggests that DUBs and other ubiquitin-related genes can be considered a very important group of genes involved in the developmental competence of the egg in sea bass and should be considered important candidates for the overall transcriptomic profile of a “good” egg.

Both microarray and qPCR analysis revealed significant differences between the groups of low and high egg quality in terms of abundance of mRNA of interferon regulatory factor 7 (*irf7*) and fucolectin-4 (*fucolectin*), both involved in innate immune response (Bianchet et al. 2002; Mommens et al. 2014). Interestingly, *irf7* and another gene related with immune response (mhc class II antigen alpha chain—*mhc2a*) were found to be very important candidate markers of egg quality in Atlantic halibut (Mommens et al. 2014). Therefore, it may be suggested that the genes related with the immune system (including *irf7* and *fucolectin*) may constitute good candidates for molecular markers of egg quality.

To the best of our knowledge, the remaining successfully annotated genes [*polk*, BTB/POZ domain-containing protein KCTD12 (*kctd12*), glucosylceramidase (*gba*), centromere protein F (*cenpf*), mitochondrial TSFM Ts translation elongation factor (*tsfm*), translational activator GCN1 (*gcnl1l*), dolichyl-phosphate beta-glucosyltransferase (*alg5*), and plectin-like isoform (*plec*)] have not yet been reported to be egg quality-dependent in any fish species. However, their involvement in a variety of important processes, such as DNA repair (*polk*; Lone et al. 2007) or enzymatically dependent processes (regulated by *gba*; Church et al. 2004; Beavan et al. 2015), indicates their possible significant contribution to the transcriptomic profile of a “good” egg. This also refers to overexpressed *cenpf* in high egg quality—a gene encoding one of the centromere proteins and involved in the cell division process—with a biological function similar to the other centromere protein (centromere protein k—*cenpk*) found to be upregulated in low egg quality in striped bass (Chapman et al. 2014). This allows the suggestion that genes involved in physical cell division (such as *cenpf* and *cenpk*) should be more closely studied in terms of their contribution to egg quality-dependent transcriptomic profile.

One of the very interesting genes overexpressed in the high egg quality group, as revealed by microarray (MA) and qPCR, is *plec*. This gene encodes plectin, which is a very large (>500 kDa) protein and an important cytolinker able to interact with a variety of cytoskeletal structures (Ackerl et al. 2007; Wiche and Winter 2011). In mice, the knockout of plectin (namely *plec1*) led to early lethality just after birth with signs of starvation and growth retardation (Ackerl et al. 2007). In humans, plectin (*PLEC1*) deficiency led to muscular dystrophy and pyloric atresia (Natsuga et al. 2010). Interestingly, the expression levels of the genes involved in cytoskeleton organization have already been found to be egg quality-dependent in rainbow trout (Bonnet et al. 2007). Considering that the assembly of the cytoskeleton also often involves membrane proteins (Herrmann and Aebi 2000), the importance of membrane protein (*mem-prot*), found to be overexpressed in high egg quality, could be additionally highlighted. This suggests that *plec*, together with other structural proteins (including *mem-prot*), may be considered in future studies on the profiling of the molecular “picture” of a “good” egg.

In the overall transcriptomic profile of the egg, genes responsible for the translational processes were reported to be very important elements securing proper embryonic development (Lanes et al. 2013; Sullivan et al. 2015). Therefore, MA analysis in our study revealed three genes (*tsfm*, *gcnl1l*, *alg5*) which have direct involvement in the translational process, and for the first time, it is suggested that they have a relation with egg quality and may constitute a good basis for the determination of the transcriptomic profile of a “good” egg. However, further investigation is still needed.

### Conclusions

In our study, we have presented for the first time data on the transcriptomic profiling of sea bass eggs characterized by different quality. We confirmed the relevance of some of the genes already reported to be potential molecular quality determinants (mainly *rnf213* and *irf7*), but we also found new genes (mainly *usp5*, *mem-prot*, *plec*, *cenpf*), whose expression patterns were not reported to be quality-dependent in any fish species. Together, our results stress the importance of genes, or groups of genes, being involved in protein ubiquitination, translation, DNA repair, and cell structure and architecture, probably the mechanisms that contribute to egg developmental competence in sea bass. This study also indicates the importance of further genomic research (for different species and with high numbers of samples characterized by a high variation in egg quality) in order to verify whether these processes are involved in the determination of egg quality competence in other species and in other culture environments, which may be an important contribution for both science and aquaculture. Additionally, it must be emphasized that there is still a high number of uncharacterized genes (with unknown ontology) that may offer a very important contribution to our knowledge. Therefore, the efforts undertaken for the characterization of these genes will have significant importance for future studies. However, apart from many unanswered questions remaining, and new questions arising, the findings of our study may contribute to a better understanding of the molecular mechanisms behind egg quality in teleosts and may help in the determination of future research priorities for the determination of the molecular profile of a “good” egg.
